# News and Events of the Week

**Published:** 1909-12-11

**Authors:** 


### News and Events of the Week.

Dr. G. H. Lodge, President of the Rotherham and District Association of Pharmacists, has been appointed a Justice of the Peace. Dr. Lodge is a licensed pharmacist, a dentist, and was registered as a medical practitioner in 1893. He has been actively associated in the municipal government of Rotherham for some years, and was Mayor of the Borough two years ago.

---

The Christmas number of *Truth* is a witty and whimsical compilation of topical "Ingoldsby Legends" which, although it cannot compare with Barham's original version either in variety of interest or in facility of rhymes, will yet be a source of delight to all readers no matter what their politics may be. Of medical interest is the amusing "Legend of Pirplepillus, the Leech," while journalists will find even more food for smiles in the ludicrous "Legend of the Carmelite Friars."

---

The General Medical Council at its last meeting considered the case of Mr. Frederick Morrish Pierce, registered as M.R.C.S.Eng., 1867, Lic.Soc.Apoth.Lond., 1867, Lic.R.Coll.Phys.Lond., 1868, M.D., Q.Univ., Irel., 1868, now of 50 Gordon Square, W.C., who had been summoned to appear before the Council on a charge that, being a certifying surgeon under the Factory and Workshop Act, 1901, he had for the purpose of evading the conditions under which he was appointed knowingly and wilfully employed another registered medical practitioner to perform his duties, and that, in order to carry out this system of evasion, he had committed certain offences, such as allowing Dr. Burgess to conduct examinations for him and sign his name to certificates that he had personally examined certain persons on certain dates, and that they were fit to perform the work on which they were engaged. It was alleged that he had signed certificates in advance, and that visits had been paid by Dr. Burgess at times when he was not Mr. Pierce's properly appointed deputy. After evidence had been given, Mr. T. P. Griffithes, solicitor, addressed the Council on behalf of Mr. Pierce. He said that until the present occasion Mr. Pierce had never had a charge of unprofessional or dishonourable conduct made against him. He had, no doubt, committed a breach of the rules in employing Dr. Burgess without getting him properly appointed, but Dr. Burgess was a fully qualified man. The Council judged Mr. Pierce to have been guilty of infamous conduct in a professional respect, and directed the Registrar to erase his name from the Medical Register.

---

An interesting note in answer to a correspondent's inquiry regarding the "composition of Dr. Jekyll's powder" appears in "The Chemist and Druggist." Our contemporary remarks that Robert Louis Stevenson was a personal friend of the late Dr. Bellyse Baildon, Ph.C. Stevenson, when at Hyères, was accustomed to go into Powell's Pharmacy and sit in the back parlour smoking cigarettes and chatting about drugs and things of that nature—always being on the look-out for local colouring. The original of Dr. Jekyll is supposed to have been Deacon Brodie of Edinburgh, who figures in "Kay's Portraits." Stevenson, like most other writers, was well aware that under the influence of certain drugs (including alcohol) men do base things which in their normal conditions they would not think of doing, and it has been suggested to us that he had the action of cannabis indica in mind when he wrote.

The Royal College of Surgeons has now collected into one room the various exhibits of historical interest which were formerly scattered throughout the museum. Among them are the mummied body of a boy who died of plague in 1665, found in the crypt of St. Botolph's old church; the skin of the heads of three Macas Indians from Ecuador, prepared so as to contract to the size of a doll's head; the boots, shoes, and gloves worn by the Irish giant O'Brien; a chain and belt used to secure lunatics; and a cast of the head of Deeming, the murderer, who was hanged in 1892.

---

The Seventeenth International Congress of Medicine, London, 1913.—A meeting of the National Committee for Great Britain and Ireland of the International Congress of Medicine was held at the rooms of the Medical Society of London on Thursday, November 26. Dr. F. W. Pavy, F.R.S. (President) in the chair. There were present: Sir William Church, K.C.B., Sir Thomas Barlow, Bart., Mr. H. T. Butlen, President of the Royal College of Surgeons of England, Sir Dyce Duckworth, Sir John Tweedy, Sir Malcolm Morris, Sir Shirley Murphy; Professor Osler, Dr. Perrier, Deputy-Inspector-General Johnston, R.N., Dr. Gordon Dill, Dr. Herringham, Dr. Gerrod, Dr. Donald Hood, Dr. Goodhart, Mr. W. H. H. Jessop, Dr. St. Clair Thomson, Mr. Hunter Tod, Mr. Eliot Creasy, the Hon. Secretaries (Dr. Clive Riviere and Mr. D'Arcy Power), and others. Mr. D'Arcy Power read a report on the Sixteenth International Congress of Medicine which was held at Budapest at the end of August. The British contingent numbered 34, and the meeting was said to have been very successful and to reflect great credit on the Executive Committee. The Congress had decided to appoint a permanent committee consisting of the Presidents, the Secretaries, and some members of the various international committees. The Permanent Committee was not to number more than 50 members; there was to be a secretary paid out of the funds of each forthcoming congress, and Dr. Pavy was appointed the first President. The offices of the Permanent Committee were to be at The Hague. Dr. Pavy notified to the meeting the acceptance of the invitation of the National Committee for Great Britain and Ireland, with the approval of his Majesty's Government, to hold the Seventeenth International Congress in London in the year 1913. It was thereupon proposed by Sir John Tweedy, seconded by Sir Dyce Duckworth, and carried unanimously that a general committee be formed to undertake the organisation required for the meeting of the Congress in London. It was further proposed by Sir William Church, seconded by Mr. Butlin, President R.C.S.(Eng.), and carried, that the National Committee for Great Britain and Ireland be dissolved and immediately reconstituted as the General Committee for this purpose, the General Committee having power to add to its numbers. It was decided that the first meeting of the General Committee should be held in February next, the officers of the National Committee holding their present positions until new officers were nominated and elected. Professor Osler moved that it be an instruction of the General Committee to see that the Colonies are adequately represented in all matters connected with the Seventeenth International Congress of Medicine. The meeting ended with a vote of thanks to the Medical Society of London for the use of the meeting-room. Mr. D'Arcy Power and Dr. Clive Riviere will be glad to receive the names of those who are interested in the forthcoming Congress as soon as possible.

